# High levels of serum vitamin D are associated with a decreased risk of metabolic diseases in both men and women, but an increased risk for coronary artery calcification in Korean men

**DOI:** 10.1186/s12933-016-0432-3

**Published:** 2016-08-12

**Authors:** Ki-Chul Sung, Yoosoo Chang, Seungho Ryu, Hye-Kyung Chung

**Affiliations:** 1 Division of Cardiology, Department of Medicine, Kangbuk Samsung Hospital, Sungkyunkwan University School of Medicine, Seoul, 110-746 South Korea; 2 Department of Occupational and Environmental Medicine, Sungkyunkwan University School of Medicine, Seoul, South Korea; 3 Severance institute for vascular and metabolic research, Yonsei University College of Medicine, Seoul, 120-749 South Korea

**Keywords:** Serum vitamin D, Metabolic diseases, Coronary artery calcification, Gender difference

## Abstract

**Background:**

There are conflicting results for relationships between serum vitamin D levels and metabolic diseases. The aim of this study was to investigate whether serum vitamin D levels were associated with various metabolic diseases including insulin resistance (IR), metabolic syndrome (MS), fatty liver (FL), and coronary artery calcification (CAC), along with assessing gender differences for these relationships in Korean adults.

**Methods:**

A total of 180,918 subjects (98,412 men and 82,506 women) who participated in a comprehensive health examination in the 2012–2013 period at Kangbuk Samsung Hospital, College of Medicine, Sungkyunkwan University were included. Serum vitamin D and metabolic markers were analyzed and CAC was estimated. Subjects were divided according to quartile groups of serum vitamin D. To examine the relationships of serum vitamin D to metabolic diseases and metabolic factors, multivariate logistic analysis was conducted.

**Results:**

High levels of serum vitamin D was associated with lower ORs for MS, IR and FL both in men and women (all p < 0.05). For men, ORs for CAC were significantly higher in third and the highest quartile groups for serum vitamin D in all the analyzed models (all p < 0.05). However, women showed no significant results between serum vitamin D and CAC.

**Conclusions:**

High levels of serum vitamin D were associated with lower risk of MS, IR and FL in both Korean men and women, but were associated with higher risk of CAC only in men, and not in women.

**Electronic supplementary material:**

The online version of this article (doi:10.1186/s12933-016-0432-3) contains supplementary material, which is available to authorized users.

## Background

It has generally been accepted that a major role of vitamin D is to maintain bone health by promoting the calcium absorption from intestine [1]. Recently, as vitamin D receptors are found in almost all human cells [2], diverse physiological roles of vitamin D besides skeletal function have been speculated. Accumulating data has suggested a potential effect of vitamin D on various chronic diseases. Also vitamin D insufficiency has become a public health concern for people having an indoor lifestyle and avoiding enough to direct sunlight. A recent study has also suggested that vitamin D deficiency is a pandemic and vitamin D deficiency, defined as having serum levels of lower than 20 ng/mL (50 nmol/L), was in 40.4 % of Europeans [3]. Previous study with data from the Korea National Health and Nutrition Examination Survey (KNHANES) reported that 48 % of Korean men and 65 % of Korean women aged over 50 years had inadequate levels of serum vitamin D [4]. Nevertheless, vitamin D insufficiency is still not treated as a serious concern, and there is a need to investigate the health benefits of vitamin D in preventing and combating relevant diseases.

25-Hydroxy vitamin D (here on referred to as serum vitamin D) is widely used as a representative marker for the whole body vitamin D status since it has a longer biological half-life and a higher concentration in comparison to 1,25-dihydroxy vitamin D, the most active form of vitamin D, and is an indicator of both endogenous and exogenous vitamin D levels [5]. Numerous studies have tried to find a relationship between levels of serum vitamin D and prevalence of various metabolic diseases [6–8]. Some results have shown that low levels of serum vitamin D are associated with increasing risk of metabolic diseases such as obesity, hypertension (HTN), insulin resistance (IR), hyperlipidemia, inflammation and cardiovascular disease (CVD) [6–8]. Other studies, however, have failed to demonstrate these benefits. As such, there is controversy as to whether vitamin D has a protective role for various metabolic diseases and vitamin D supplementation is absolutely necessary. It should be noted that most previous studies have had the limitation of using data from a relatively small number of subjects and also neglected potential gender differences and possible multiple confounding factors which affect serum vitamin D [6, 8]. Therefore, a large-scale study also considering gender differences and potential multiple confounding factors is required to find the associations between serum vitamin D levels and occurrence of metabolic diseases. The aim of this study was to examine the distribution of vitamin D levels in Korean adults. We also intended to investigate whether serum vitamin D levels were associated with diverse metabolic conditions such as insulin resistance, metabolic syndrome (MS), fatty liver (FL) and coronary artery calcification (CAC) with respect to gender and using a large-scale dataset from occupational cohort in Korean adults.

## Subjects and methods

### Study population

**Fig. 1:**
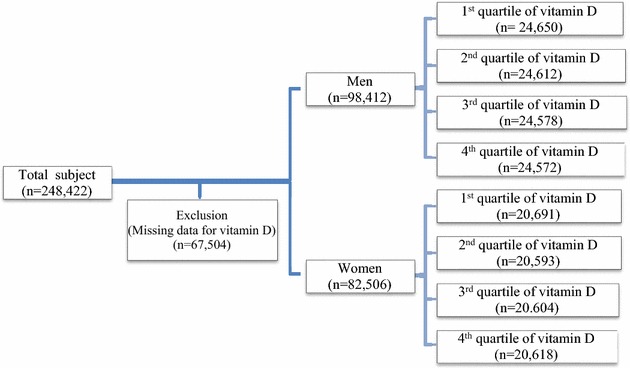
The flow diagram of this study

The study population consisted of individuals who participated in a comprehensive health examination in 2012–2013 at Kangbuk Samsung Hospital, College of Medicine, Sungkyunkwan University, Korea. For 80 % of the subjects, they had employment. Initially 248,422 subjects were included. After excluding 67,504 subjects whose values for vitamin D levels were missing, a total of 180,918 subjects (98,412 men and 82,506 women) were included for the final analysis (Fig. 1). In South Korea, employees are required to participate in annual or biannual health examinations as mandated by the Industrial Safety and Health Law. Some subjects voluntarily pay and in other instances, employers pay for these health evaluations. The Institutional Review Board (IRB) at Kangbuk Samsung Hospital approved the study, and no specific informed consent from the subjects was considered to be necessary.

### Anthropometric measurements and general characteristics

Body mass index (BMI) was calculated as weight in kilograms divided by height in meters squared. Waist circumference was measured according to a standardized operating protocol. Briefly, the mid point between the lowest rib and the superior iliac crest was identified in the mid-axillary line. At this point a measuring tape (SECA 200, circumference measuring tape) was placed around the abdomen, ensuring that the tape was horizontal to the floor. A measurement was taken to the nearest 0.1 cm, and at the end of a normal expiration. This measurement was then repeated. If the two readings varied by more than 1 %, there was a computer-generated prompt to take a third reading. Questionnaires were used to ascertain information regarding alcohol consumption (glasses/day), smoking (never smoked, ex-smoker, current smoker), and frequency of moderate activity or exercise per week (moderate activity was defined as more than 30 min activity per day that induced slight breathlessness).

### Medical history and diagnosis

Prior history of CVD, HTN, and diabetes mellitus (DM) was obtained by questionnaires. HTN was defined by systolic blood pressure ≥140 mmHg or diastolic blood pressure ≥90 mmHg, or being on medication for HTN. DM was diagnosed according to criteria of the American Diabetes Association as either fasting glucose ≥126 mg/dL, HbA1C ≥6.5 %, or use of an oral hypoglycemic agent. Abdominal ultrasonography (Logic Q700 MR, GE Healthcare, Tokyo, Japan) with a 3.5-MHz probe was performed for all participants by experienced clinical radiologists, and FL was diagnosed on the basis of standard criteria including hepatorenal echo contrast, liver brightness and vascular blurring. Fatty infiltration was classified as an increase in echogenicity of the liver compared with that of the renal cortex where the diaphragm and intrahepatic vessels appeared healthy [9]. All computed tomography scans (CT scan) were obtained with a Light speed VCT XTe-64 slice MDCT scanner (GE Healthcare) with the same standard scanning protocol using 40*2.5-mm section collimation, 400 ms rotation time, 120 kV tube voltage, and 124 mAS (310 mA*0.4 s) tube current under ECG-gated dose modulation. The quantitative CAC scores were calculated according to the method described by Agatston et al. [10]. MS was defined by the 2009 joint interim statement of the International Diabetes Federation Task Force on Epidemiology and Prevention criteria, with waist circumference thresholds of ≥90 cm for men and ≥80 cm for women that are specific for Asian populations [11]. Same criteria for waist circumference were used for definition of abdominal obesity.

### Biochemical markers and serum vitamin D

Blood samples were collected after an overnight fast. Serum total cholesterol, triglycerides and uric acid were determined using an enzymatic colorimetric assay. Low-density lipoprotein cholesterol (LDL-C) and high-density lipoprotein cholesterol (HDL-C) were directly measured using a homogeneous enzymatic colorimetric assay. Serum high sensitivity C-reactive protein (hs-CRP) was determined using a particle-enhanced immunoturbidimetric assay on a Modular Analytics P800 apparatus (Roche Diagnostics, Rotkreuz, Switzerland). Serum fasting glucose concentrations were measured using the hexokinase method on a Cobas Integra 800 apparatus (Roche Diagnostics). Serum insulin was measured using an electrochemiluminescence immunoassay on a Modular Analytics E170 apparatus (Roche Diagnostics). IR was measured using the homeostatic model assessment of insulin resistance (HOMA-IR) and was obtained by applying the following formula: HOMA-IR = fasting insulin (IU/mL) × fasting blood glucose (mmol/L)/22.5 [12]. To assess serum vitamin D status, total vitamin D (25-OH) was measured with a competitive immunoassay using the Elecsys vitamin D (25-OH) total assay on Modular E170 immunoanalyzer (Roche, Basel, Switzerland).

### Statistical analysis

General characteristics and prevalence of metabolic diseases of the study participants were compared among quartile groups of serum vitamin D. Categorical variables are presented as number (%). Continuous variables are presented as mean (SD) or median (IQR) based on the distribution of the data. Differences across quartile groups were tested using Chi square tests or ANOVA tests. The distribution of continuous variables was evaluated, and right-skewed variables were log transformed for the one-way analysis of variance. To test for linear trends, we included the median value of each category as a continuous variable in the regression model. To evaluate the associations of outcome variables and quartiles of serum vitamin D, we used a logistic regression model estimating the odds ratios (ORs) with 95 % confidence intervals (CIs) for metabolic diseases including CAC. We used two models to progressively adjust for potential confounders. Model 1 was adjusted for age. Model 2 was adjusted for age, year of screening exam, center, smoking, alcohol, education, physical activity and season of the year. For CAC, Models 3 and 4 were constructed in addition to Models 1 and 2. Model 3 was additionally adjusted for HTN, DM and CVD plus Model 2, and Model 4 was additionally adjusted for BMI, SBP, glucose and LDL-C plus Model 3. Statistical analyses were performed with Stata version 11.2 (StataCorp LP, College Station, TX, USA). All reported *p* values are 2-tailed, and comparisons with p < 0.05 were considered statistically significant.

## Results

### General characteristics including the metabolic markers of the subjects

General characteristics and metabolic markers of subjects by gender are presented in Tables 1 and 2. For the male subjects, the mean age was 39.8 ± 8.1 years and BMI was 24.5 ± 3.0 kg/m^2^. Of the 98,412 male subjects, 39.4 % were obese and 42.0 % had fatty liver. When general characteristics and metabolic markers were compared according to quartile groups of serum vitamin D levels, most of the markers except for TG were significantly different (all p < 0.05). For female subjects, the mean age was 38.5 ± 8.7 years and BMI was 21.7 ± 3.1 kg/m^2^. Of the 82,506 female subjects, 27.4 % showed abdominal obesity and 11.8 % had FL. Most of the markers except for systolic blood pressure (SBP), diastolic blood pressure (DBP), glucose and TG were significantly different among the quartile groups for serum vitamin D (all p < 0.05).

**Table 1:** General characteristics by quartiles of serum vitamin D levels in men (n = 98,412)

Characteristics	Total subjects	Quartiles of serum vitamin D levels				*p* for trend
		Q1 (<12.9 mg/dL)	Q2 (12.9–16.6 mg/dL)	Q3 (16.7–21.4 mg/dL)	Q4 (≥21.4 mg/dL)	
		n = 24,650	n = 24,612	n = 24,578	n = 24,572	
Age (years)	39.8 (8.1)	37.9 (7.6)	39.0 (7.7)	40.0 (7.7)	42.1 (8.8)	<0.001
BMI (kg/m^2^)	24.5 (3.0)	24.3 (3.2)	24.5 (3.0)	24.6 (2.9)	24.5 (2.9)	<0.001
Current smoker (%)	36.0	32.4	34.5	36.9	40.2	<0.001
Alcohol intake (%)^a^	33.2	26.0	30.8	35.7	40.4	<0.001
Regular exercise (%)^b^	14.4	11.3	13.3	14.9	18.0	<0.001
Higher education (%)^c^	85.5	86.8	87.2	85.9	82.2	<0.001
SBP (mmHg)	112.8 (11.3)	112.2 (11.1)	112.6 (11.2)	112.9 (11.2)	113.3 (11.4)	<0.001
DBP (mmHg)	73.7 (9.4)	73.1 (9.3)	73.5 (9.4)	73.9 (9.4)	74.2 (9.4)	<0.001
Glucose (mg/dL)	97.2 (15.1)	96.5 (15.1)	97.0 (15.0)	97.5 (15.6)	97.9 (14.7)	<0.001
Insulin (μU/mL)^d^	5.75 (3.86–8.37)	5.92 (3.97–8.63)	5.82 (3.91–8.44)	5.77 (3.88–8.39)	5.47 (3.71–8.0)	<0.001
TC (mg/dL)	199.2 (34.2)	196.3 (34.3)	199.3 (34.1)	201.0 (34.3)	200.3 (34.1)	<0.001
LDL-C (mg/dL)	126.9 (31.1)	124.5 (31.0)	127.1 (30.8)	128.6 (31.3)	127.5 (31.2)	<0.001
HDL-C (mg/dL)	52.2 (12.5)	51.5 (12.4)	51.9 (12.2)	52.4 (12.5)	53.1 (12.8)	<0.001
TG (mg/dL)^d^	114 (80–163)	112 (79–162)	113 (81–164)	115 (82–165)	114 (81–162)	0.070
hs-CRP (mg/L)^d^	0.5 (0.3–1.1)	0.5 (0.3–1.1)	0.5 (0.3–1.1)	0.5 (0.3–1.1)	0.5 (0.3–1.1)	0.013
Obesity (%)	39.4	36.2	39.9	41.3	40.4	<0.001
Abdominal obesity (%)	30.2	28.9	30.8	31.3	30.1	0.001
DM (%)	4.59	3.81	4.25	4.78	5.54	<0.001
HTN (%)	14.8	12.7	13.5	15.3	17.7	<0.001
FL (%)	42.0	42.4	42.9	42.9	39.8	<0.001
MS (%)	15.3	14.5	15.4	15.7	15.5	0.001
CVD (%)	1.19	1.00	0.98	1.21	1.56	<0.001
HOMA-IR^d^	1.36 (0.90–2.03)	1.40 (0.91–2.07)	1.38 (0.90–2.05)	1.37 (0.90–2.05)	1.31 (0.86–1.97)	<0.001

Data are expressed as mean (SD)

*BMI* body mass index, *SBP* systolic blood pressure, *DBP* diastolic blood pressure, *TC* total cholesterol, *HDL-C* HDL cholesterol, *TG* triglyceride, *hs-CRP* high sensitive C-reactive protein, *DM* diabetes mellitus, *HTN* hypertension, *FL* fatty liver, *MS* metabolic syndrome, *CVD* cardiovascular disease, *HOMA-IR* homeostasis model assessment of insulin resistance

^a^≥20 g/day, ^b^ ≥ 3 times/week, ^c^ ≥college graduate, ^d^ data are expressed as median (IQR)

**Table 2:** General characteristics by quartiles of serum vitamin D levels in women (n = 82,506)

Characteristics	Total subjects	Quartiles of serum vitamin D levels				*p* for trend
		Q1 (<9.7 mg/dL)	Q2 (9.7–12.8 mg/dL)	Q3 (12.8–17.2 mg/dL)	Q4 (≥17.2 mg/dL)	
		n = 20,691	n = 20,593	n = 20,604	n = 20,618	
Age (years)	38.5 (8.7)	37.8 (8.6)	37.9 (8.3)	38.3 (8.3)	40.0 (9.4)	<0.001
BMI (kg/m^2^)	21.7 (3.1)	21.7 (3.3)	21.8 (3.2)	21.7 (3.0)	21.6 (2.8)	<0.001
Current smoker (%)	2.38	2.70	2.45	2.07	2.29	0.002
Alcohol intake (%)^a^	6.20	5.51	6.23	6.29	6.76	<0.001
Regular exercise (%)^b^	12.8	10.2	11.7	13.3	16.1	<0.001
Higher education (%)^c^	71.6	68.4	71.1	73.3	73.8	<0.001
SBP (mmHg)	100.0 (11.0)	100.1 (10.9)	100.0 (10.9)	99.8 (10.9)	100.0 (11.2)	0.357
DBP (mmHg)	64.4 (8.5)	64.3 (8.4)	64.5 (8.5)	64.3 (8.5)	64.5 (8.6)	0.053
Glucose (mg/dL)	91.4 (11.6)	91.4 (11.9)	91.4 (11.4)	91.2 (11.2)	91.5 (11.8)	0.754
Insulin (μU/mL)^d^	4.81 (3.29–6.84)	4.96 (3.42–7.06)	4.92 (3.38–7.02)	4.79 (3.27–6.84)	4.55 (3.13–6.43)	<0.001
TC (mg/dL)	186.9 (32.6)	184.6 (32.1)	187.0 (32.8)	187.7 (32.5)	188.6 (32.8)	<0.001
LDL-C (mg/dL)	109.3 (29.6)	107.9 (29.0)	109.5 (29.9)	109.9 (29.6)	110.1 (29.7)	<0.001
HDL-C (mg/dL)	64.3 (14.5)	63.1 (14.2)	64.1 (14.5)	64.5 (14.6)	65.4 (14.8)	<0.001
TG (mg/dL)^d^	70 (54–95)	69 (53–95)	70 (54–95)	70 (53.5–95)	70 (54–96)	0.061
hs-CRP (mg/L)^d^	0.3 (0.2–0.7)	0.3 (0.2–0.7)	0.3 (0.2–0.7)	0.3 (0.2–0.7)	0.3 (0.2–0.7)	<0.001
Obesity (%)	13.0	13.7	13.7	13.1	11.3	<0.001
Abdominal obesity (%)	27.4	27.4	28.2	27.8	26.3	0.014
DM (%)	1.72	1.59	1.59	1.56	2.13	<0.001
HTN (%)	5.23	5.03	4.61	4.85	6.42	<0.001
FL (%)	11.8	12.3	11.8	11.8	11.1	0.001
MS (%)	5.6	5.8	5.8	5.4	5.2	0.001
CVD (%)	0.68	0.57	0.63	0.66	0.86	0.003
HOMA-IR^d^	1.08 (0.72–1.57)	1.11 (0.75–1.62)	1.10 (0.73–1.61)	1.07 (0.71–1.57)	1.02 (0.68–1.49)	<0.001

Data are expressed as mean (SD)

*BMI* body mass index, *SBP* systolic blood pressure, *DBP* diastolic blood pressure, *TC* total cholesterol, *HDL-C* HDL cholesterol, *TG* triglyceride, *hs-CRP* high sensitive C-reactive protein, *DM* diabetes mellitus, *HTN* hypertension, *FL* fatty liver, *MS* metabolic syndrome, *CVD* cardiovascular disease, *HOMA-IR* homeostasis model assessment of insulin resistance

^a^≥20 g/day, ^b^ ≥3 times/week, ^c^ ≥college graduate, ^d^ data are expressed as median (IQR)

### Distribution of serum vitamin D levels

**Fig. 2:**
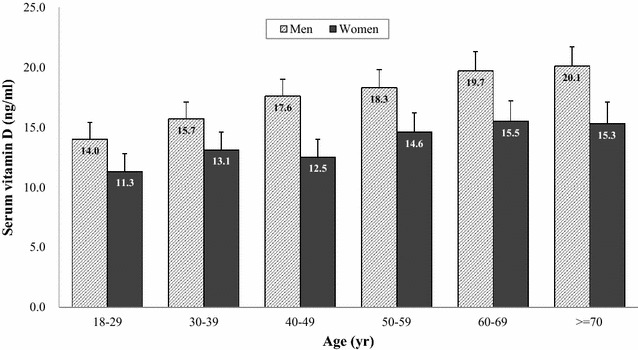
The means (SD) of serum vitamin D levels according to each age group in men and women

**Fig. 3:**
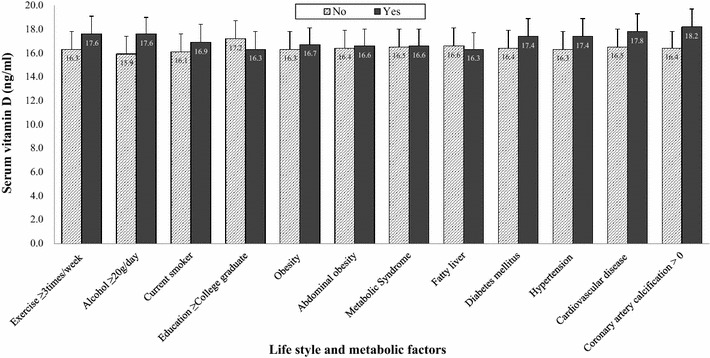
The means (SD) of serum vitamin D levels according to life style and metabolic factors in men

**Fig. 4:**
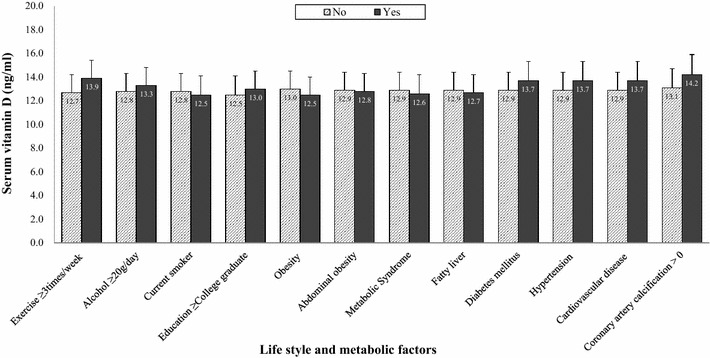
The means (SD) of serum vitamin D levels according to life style and metabolic factors in women

Figure 2 indicates the distribution of serum vitamin D levels according to each age group in the men and women groups. For men, the levels of serum vitamin D in each age group showed a trend of increasing with age. Men subjects over 70 had the highest mean of serum vitamin D levels. In addition, the levels of serum vitamin D in men were higher than those of women for all age groups (all p < 0.05). For female subjects, women in their 60 s showed the highest means of serum vitamin D levels. Figures 3 and 4 suggest the distribution of serum vitamin D levels according to life styles and metabolic factors in men and women subjects, respectively (all p < 0.05 between men and women).

### Odds ratios for metabolic diseases and metabolic factors by quartiles of serum vitamin D

To examine the relationships of serum vitamin D to metabolic diseases and metabolic factors, multivariate logistic analysis was conducted. The ORs for metabolic diseases and metabolic factors by quartile groups of serum vitamin D are presented in Tables 3 and 4. For men, the ORs for HTN were significantly higher in third and the highest quartile group of serum vitamin D in Model 1 (OR 1.08, 95 % CI 1.03–1.14, OR 1.09, 95 % CI 1.03–1.15, respectively), but these significances disappeared in Model 2. ORs for DM were significantly lower in highest quartile group only in Model 2 (OR 0.88, 95 % CI 0.79–0.98). ORs for FL and IR (HOMA-IR >75 percentile) were also significantly lower in the highest quartile group for both models (OR 0.83, 95 % CI 0.80–0.86, OR 0.86, 95 % CI 0.83–0.90 in Model 1, OR 0.77, 95 % CI 0.74–0.80, OR 0.84, 95 % CI 0.80–0.87 in Model 2). ORs for MS were lower in third and the highest quartile group only in Model 2 (OR 0.92, 95 % CI 0.87–0.97, OR 0.81, 95 % CI 0.76–0.86). ORs for abdominal obesity were higher in second, third and the highest quartile groups in Model 1, and higher in second quartile group in Model 2. For women, the ORs for HTN were significantly lower in the highest quartile group of serum vitamin D in both models (OR 0.85, 95 % CI 0.78–0.94 in Model 1, OR 0.84, 95 % CI 0.74–0.95 in Model 2). ORs for FL, MS and IR (HOMA-IR >75 percentile) were also significantly lower in third and the highest quartile groups in both models (OR 0.74, 95 % CI 0.69–0.78, OR 0.71, 95 % CI 0.65–0.78, OR 0.73, 95 % CI 0.69–0.77 in Model 1, OR 0.69, 95 % CI 0.64–0.75, OR 0.65, 95 % CI 0.58–0.73, OR 0.74, 95 % CI 0.69–0.78 in Model 2). However, the ORs for high hs-CRP (>1.0 mg/L) was higher in second and the highest quartile groups for both models (OR 1.08, 95 % CI 1.03–1.14, OR 1.08, 95 % CI 1.03–1.14 in Model 1, OR 1.09, 95 % CI 1.02–1.15, OR 1.11, 95 % CI 1.05–1.18 in Model 2).

**Table 3:** ORs (95 % CIs) for metabolic disease and metabolic factors by quartiles of serum vitamin D levels in men (n = 98,412)

Characteristics	Quartiles of serum vitamin D levels				*p* for trend
	Q1 (< 12.9 mg/dL)	Q2 (12.9–16.6 mg/dL)	Q3 (16.7–21.4 mg/dL)	Q4 (≥21.4 mg/dL)	
	n = 24,650	n = 24,612	n = 24,578	n = 24,572	
HTN (%)					
Model 1	1.00 (reference)	1.00 (0.95–1.06)	1.08 (1.03–1.14)	1.09 (1.03–1.15)	<0.001
Model 2	1.00 (reference)	0.98 (0.92–1.04)	1.01 (0.96–1.07)	1.02 (0.96–1.08)	0.371
DM (%)					
Model 1	1.00 (reference)	1.03 (0.94–1.13)	1.06 (0.97–1.17)	0.96 (0.88–1.05)	0.447
Model 2	1.00 (reference)	0.99 (0.89–1.10)	0.98 (0.88–1.09)	0.88 (0.79–0.98)	0.011
CVD (%)					
Model 1	1.00 (reference)	0.89 (0.74–1.07)	1.02 (0.86–1.21)	0.97 (0.82–1.14)	0.901
Model 2	1.00 (reference)	0.90 (0.73–1.11)	0.99 (0.81–1.20)	0.94 (0.77–1.14)	0.742
FL (%)					
Model 1	1.00 (reference)	1.00 (0.97–1.04)	0.98 (0.95–1.02)	0.83 (0.80–0.86)	<0.001
Model 2	1.00 (reference)	0.97 (0.93–1.01)	0.92 (0.89–0.96)	0.77 (0.74–0.80)	<0.001
MS (%)					
Model 1	1.00 (reference)	1.05 (0.99–1.10)	1.04 (0.99–1.09)	0.95 (0.90–1.00)	0.045
Model 2	1.00 (reference)	0.99 (0.94–1.04)	0.92 (0.87–0.97)	0.81 (0.76–0.86)	<0.001
Abdominal obesity (%)					
Model 1	1.00 (reference)	1.09 (1.05–1.13)	1.11 (1.07–1.16)	1.05 (1.01–1.09)	0.011
Model 2	1.00 (reference)	1.07 (1.02–1.11)	1.04 (1.00–1.09)	0.96 (0.92–1.00)	0.031
HOMA-IR (%)^a^					
Model 1	1.00 (reference)	0.97 (0.93–1.01)	0.97 (0.94–1.01)	0.86 (0.83–0.90)	<0.001
Model 2	1.00 (reference)	0.97 (0.93–1.01)	0.96 (0.92–1.00)	0.84 (0.80–0.87)	<0.001
hs-CRP (%)^b^					
Model 1	1.00 (reference)	1.00 (0.96–1.04)	1.01 (0.97–1.05)	0.99 (0.95–1.03)	0.859
Model 2	1.00 (reference)	1.01 (0.96–1.05)	1.00 (0.96–1.04)	0.98 (0.93–1.02)	0.270

Differences were tested using multivariate-adjusted logistic regression analysis

Model 1: adjustment for age, Model 2: adjustment for model 1 plus year of screening exam, center, smoking, alcohol, education, physical activity and season

*ORs* odds ratios, *CIs* confidence intervals, *HTN* hypertension, *FL* fatty liver, *MS* metabolic syndrome, *CVD* cardiovascular disease, *HOMA-IR* homeostasis model assessment of insulin resistance, *hs-CRP* high sensitive C-reactive protein

^a^HOMA-IR >75 percentile, ^b^ hs-CRP > 1.0 mg/L

**Table 4:** ORs (95 % CIs) for metabolic disease and metabolic factors by quartiles of serum vitamin D levels in women (n = 82,506)

Characteristics	Quartiles of serum vitamin D levels				*p* for trend
	Q1 (<9.7 mg/dL)	Q2 (9.7–12.8 mg/dL)	Q3 (12.8–17.2 mg/dL)	Q4 (≥17.2 mg/dL)	
	n = 20,691	n = 20,593	n = 20,604	n = 20,618	
HTN					
Model 1	1.00 (reference)	0.92 (0.83–1.02)	0.92 (0.84–1.02)	0.85 (0.78–0.94)	0.002
Model 2	1.00 (reference)	0.88 (0.78–1.01)	0.89 (0.78–1.01)	0.84 (0.74–0.95)	0.010
DM					
Model 1	1.00 (reference)	1.03 (0.88–1.21)	0.96 (0.82–1.12)	0.95 (0.82–1.11)	0.373
Model 2	1.00 (reference)	1.12 (0.91–1.38)	0.94 (0.76–1.16)	0.92 (0.75–1.13)	0.198
CVD					
Model 1	1.00 (reference)	1.14 (0.88–1.47)	1.15 (0.89–1.47)	1.05 (0.83–1.34)	0.767
Model 2	1.00 (reference)	1.31 (0.92–1.85)	1.14 (0.80–1.62)	1.13 (0.80–1.59)	0.756
FL					
Model 1	1.00 (reference)	0.95 (0.89–1.01)	0.93 (0.88–0.99)	0.74 (0.69–0.78)	<0.001
Model 2	1.00 (reference)	0.93 (0.86–1.00)	0.89 (0.83–0.96)	0.69 (0.64–0.75)	<0.001
MS					
Model 1	1.00 (reference)	1.01 (0.93–1.10)	0.91 (0.83–0.99)	0.71 (0.65–0.78)	<0.001
Model 2	1.00 (reference)	0.97 (0.87–1.08)	0.84 (0.76–0.94)	0.65 (0.58–0.73)	<0.001
Abdominal obesity					
Model 1	1.00 (reference)	1.04 (1.00–1.09)	1.01 (0.96–1.05)	0.85 (0.81–0.89)	<0.001
Model 2	1.00 (reference)	1.06 (1.01–1.12)	1.00 (0.95–1.06)	0.86 (0.81–0.91)	<0.001
HOMA-IR^a^					
Model 1	1.00 (reference)	0.98 (0.94–1.03)	0.89 (0.85–0.94)	0.73 (0.69–0.77)	<0.001
Model 2	1.00 (reference)	0.97 (0.92–1.03)	0.90 (0.85–0.96)	0.74 (0.69–0.78)	<0.001
hs-CRP^b^					
Model 1	1.00 (reference)	1.08 (1.03–1.14)	1.05 (1.00–1.10)	1.08 (1.03–1.14)	0.009
Model 2	1.00 (reference)	1.09 (1.02–1.15)	1.03 (0.97–1.10)	1.11 (1.05–1.18)	0.005

Differences were tested using multivariate-adjusted logistic regression analysis

Model 1: adjustment for age, Model 2: adjustment for model 1 plus year of screening exam, center, smoking, alcohol, education, physical activity and season

*ORs* odds ratios, *CIs* confidence intervals, *HTN* hypertension, *FL* fatty liver, *MS* metabolic syndrome, *CVD* cardiovascular disease, *HOMA-IR* homeostasis model assessment of insulin resistance, *hs-CRP* high sensitive C-reactive protein

^a^HOMA-IR >75 percentile, ^b^ hs-CRP >1.0 mg/L

### Odds ratios for CAC by quartiles of serum vitamin D levels

We analyzed only for subjects who had data for CAC (number of men = 19,999; women = 5510) to confirm that relationship between serum levels of vitamin D and CAC (Additional file 1, Tables 5 and 6). Four models were constructed for logistic analysis. For men, ORs for CAC were significantly higher in third and the highest quartile groups of serum vitamin D in all models (OR 1.39, 95 % CI 1.22–1.58, OR 1.36, 95 % CI 1.20–1.54 in Model 1, OR 1.32, 95 % CI 1.15–1.53, OR 1.27, 95 % CI 1.10–1.47 in Model 2, OR 1.33, 95 % CI 1.15–1.53, OR 1.28, 95 % CI 1.11–1.48 in Model 3, OR 1.27, 95 % CI 1.10–1.47, OR 1.26, 95 % CI 1.08–1.46 in Model 4, respectively). For women, ORs for CAC were significantly lower in the highest quartile group only in Model 2 (OR 0.59, 95 % CI 0.39–0.97), but theses significances were not seen in Models 1, 3 and 4.

**Table 5:** ORs (95 % CIs) for CAC by quartiles of serum vitamin D levels in men (n = 19,999)

	Quartiles of serum vitamin D levels				*p* for trend
	Q1 (<13.0 mg/dL)	Q2 (13.0–16.8 mg/dL)	Q3 (16.8–21.5 mg/dL)	Q4 (≥21.5 mg/dL)	
	n = 5006	n = 4994	n = 5000	n = 4999	
CAC > 0					
Model 1	1.00 (reference)	1.18 (1.04–1.35)	1.39 (1.22–1.58)	1.36 (1.20–1.54)	<0.001
Model 2	1.00 (reference)	1.14 (0.98–1.32)	1.32 (1.15–1.53)	1.27 (1.10–1.47)	<0.001
Model 3	1.00 (reference)	1.14 (0.98–1.32)	1.33 (1.15–1.53)	1.28 (1.11–1.48)	<0.001
Model 4	1.00 (reference)	1.11 (0.95–1.29)	1.27 (1.10–1.47)	1.26 (1.08–1.46)	0.001

Differences were tested using multivariate-adjusted logistic regression analysis

Model 1: adjustment for age

Model 2: adjustment for model 1 plus year of screening exam, center, smoking, alcohol, education, physical activity and season

Model 3: adjustment for model 2 plus HTN, DM and CVD

Model 4: adjustment for model 3 plus BMI, SBP, glucose and LDL-C

*ORs* odds ratios, *CIs* confidence intervals, *CAC* coronary artery calcification

**Table 6:** ORs (95 % CIs) for CAC by quartiles of serum vitamin D levels in women (n = 5510)

	Quartiles of serum vitamin D levels				*p* for trend
	Q1 (<9.8 mg/dL)	Q2 (9.8–13.0 mg/dL)	Q3 (13.0–17.6 mg/dL)	Q4 (≥17.6 mg/dL)	
	n = 1380	n = 1378	n = 1378	n = 1374	
CAC > 0					
Model 1	1.00 (reference)	1.10 (0.75–1.60)	0.75 (0.51–1.11)	0.86 (0.61–1.23)	0.181
Model 2	1.00 (reference)	0.99 (0.61–1.62)	0.63 (0.38–1.06)	0.59 (0.36–0.97)	0.012
Model 3	1.00 (reference)	1.07 (0.64–1.79)	0.70 (0.41–1.19)	0.69 (0.41–1.15)	0.065
Model 4	1.00 (reference)	1.02 (0.61–1.71)	0.68 (0.40–1.17)	0.69 (0.41–1.15)	0.068

Differences were tested using multivariate-adjusted logistic regression analysis

Model 1: adjustment for age

Model 2: adjustment for model 1 plus year of screening exam, center, smoking, alcohol, education, physical activity, season

Model 3: adjustment for model 2 plus HTN, DM, CVD

Model 4: adjustment for model 3 plus BMI, SBP, glucose, LDL-C

*ORs* odds ratios, *CIs* confidence intervals, *CAC* coronary artery calcification

## Discussion

In this large occupational cohort of Korean adults, the levels of serum vitamin D were relatively low for all age groups in both men and women. We could also show that high levels of serum vitamin D were associated with lower risk of MS, IR and FL for both Korean men and women, but were associated with higher risk of CAC only in men and not in women.

As the mean of serum vitamin D at all age groups belonged to a range of mild vitamin D deficiency, a number of Koreans are suspected to be at high risk of vitamin D deficiency by generally used cut point (10–20 ng/mL) [13]. It should be stressed though that there is still no consensus on criteria for vitamin D deficiency or what the optimal levels of serum vitamin D ought to be [13]. This is because the effect of vitamin D levels on health outcomes is difficult to evaluate and the results of studies on vitamin D deficiency vary depending on cut point of low vitamin D levels and characteristics of the study population. Some of studies reported that intestinal calcium absorption could be maximally maintained even under the lower levels than 20 ng/mL of serum vitamin D [14, 15]. For the Korean population, more evidence is needed to assess relative vitamin D deficiency and the optimal levels of serum vitamin D.

When we conducted a logistic analysis, the levels of serum vitamin D was inversely associated with ORs for MS, IR and FL in both men and women. This implies that maintaining the serum vitamin D over a certain level may have a protective effect on occurrence of metabolic diseases including MS, IR and FL. These results are consistent with those of previous studies suggesting a relationship between serum vitamin D and occurrence of metabolic diseases [16–20]. A study with Korean post-menopausal women reported that a low serum vitamin D level was significantly associated with increased presence of MS [16], and another study with Malay adults also proposed that subjects with insufficient serum vitamin D showed higher ORs for MS [17]. Recent meta-analysis concluded that vitamin D status was associated with a risk for MS in cross-sectional studies [18]. Similar to our results, accumulating data has indicated that serum vitamin D have an inverse relationships with occurrence of IR, key component of MS [19, 20]. A prospective study of non-diabetic individuals demonstrated that serum vitamin D levels was inversely associated with a risk of IR [19], and a cross sectional study also reported the same results [20]. In addition, one study on severely obese subjects with newly onset abnormal glucose metabolism also proved the potential role of vitamin D in the regulation of glucose metabolism [21].

In our study, the OR for DM was lower only in the highest vitamin D group in men subjects after adjustment. Potential beneficial effects of vitamin D on the prevention or cure of DM emerged as an important interest. A large prospective study suggested that a daily supplement of vitamin D with calcium reduce the incidence of type 2 DM in women [22]. In contrast, the recent reviews of studies concluded that vitamin D supplementation has no effect on development and treatment of type 2 DM [23, 24]. Woman’s Health Initiative trial also reported that vitamin D couldn’t reduce the incidence of type 2 DM in healthy postmenopausal women [25]. Meanwhile, many studies found beneficial effects of vitamin D supplementation in early life on risk of type 1 DM development [26, 27]. Meta-analysis proved significant efficacy of vitamin D on type 1 DM [28].

Generally, individuals with a long-standing MS are also linked to a higher risk of developing FL [29]. In our study, FL showed similar results with MS, which inversely related with serum vitamin D. Meta-analysis suggested that patients with non-alcoholic fatty liver disease (NAFLD) had significantly lower levels of serum vitamin D and a higher likelihood of vitamin D deficiency [30]. Another study on adults with normal serum liver enzymes also found that low serum vitamin D were associated with the presence of NAFLD independently from MS and IR [31]. Although it is difficult to explain the mechanism on how vitamin D can affect occurrence of diverse metabolic diseases and knowing that these group of diseases are intricately linked, plausible hypotheses do exist. Low levels of vitamin D can increase parathyroid hormone levels (PTH), which can then reduce insulin sensitivity by regulating the intracellular free calcium concentrations [32]. Vitamin D also can affect IR by stimulating the expression of the insulin receptor [33]. We postulate that low levels of vitamin D may cause MS induced by the IR, and then long-term MS finally may lead to FL. Based on the above results, since vitamin D seems to have a beneficial effect for MS, IR and FL, vitamin D status should be routinely checked and more active treatment should be considered for subjects with vitamin D insufficiency or deficiency. However, as some of previous studies have failed to prove the relationship between vitamin D and metabolic diseases and the beneficial effect of vitamin D supplementation on metabolic diseases [34], a prospective study will be needed to confirm the effectiveness of vitamin D in prevention and possibly treatment of metabolic diseases.

We conducted a subgroup analysis limit for subjects with data for CAC. Contrary to our expectations, a noticeable finding was that high level of serum vitamin D was significantly associated with high ORs for CAC and only in men subjects. High level of serum vitamin D seems to have harmful effects on CVD in terms of increasing the risk of CAC. This raises the possibility that high level of serum vitamin D may be a promoter of vascular calcification and a risk factor for subclinical atherosclerosis in some cases. These results are in contrast with the relationship of vitamin D levels for occurrence of MS, IR and FL in our findings. A possible explanation for an association between vitamin D and CAC is that vitamin D can elevate the absorption of calcium and phosphorus, and the increased calcium and phosphorus load may stimulate the development of vascular calcification. Several experimental studies have shown vitamin D-associated vascular calcification in mouse models [35–37], and a clinical study also suggested that increased serum levels of calcium and phosphate induced by vitamin D supplementation may cause the vascular wall mineralization in chronic kidney disease (CKD) patients [38]. However, previous evidences of a link between serum vitamin D and occurrence of CAC showed mixed results and were inconclusive [38–41]. Some of studies also did not find an evidence to suggest a link between serum vitamin D and CAC [39, 40, 42]. Prospective study proved no relation of vitamin D with CVD complication in type 1 diabetes [43]. Other studies suggested that vitamin D deficiency dose not predict progression of CAC [38] and daily supplementation of vitamin D during 12 weeks does not affect cardiovascular risk including vascular calcification markers [44]. Whereas other studies revealed that vitamin D levels were inversely correlated with CAC [41, 45] and that vitamin D deficiency had a significant positive association with the presence of CAC [46]. Based on these conflicting results, we speculate that vitamin D may have dual, but opposing, roles in vascular calcification according to levels of serum vitamin D and characteristics of the subjects.

In our results, the relations of vitamin D to metabolic diseases may be different according to gender. An interesting finding from our study is that a positive correlation between vitamin D and CAC seemed to exist only in men, and this was not observed in women. On the contrary, serum vitamin D levels in women were inversely associated with occurrence of CAC particularly in Model 2. Men and women seem to respond differently to vitamin D levels regarding CAC, and this may be due to gender differences related to effects of vitamin D physiology and CVD [47–49], and differences from hormonal influences. These gender differences in our results may also be related to the fact that men have higher circulating levels of vitamin D and a higher prevalence of CAC than women. Some studies support our assertion. Recent study showed gender- and age-related difference between vitamin D deficiency and cardiometabolic risk factors [50]. Proteomic study also demonstrated significant modulation between men and women that mapped to pathways of vitamin D function [51].

Gender has been known to be independent predictor for levels of serum vitamin D. Previous investigations have observed that levels of serum vitamin D is higher in men than women, and female gender itself is one of the risk factors for hypovitaminosis D [48]. One possible explanation for this difference between men and women is that the intestinal calcium absorption may be more vitamin D-dependent in men than in women [49]. In addition, the sex hormones like estrogen which interact with vitamin D could be responsible for the differences in calcium absorption under the same vitamin D conditions. Meanwhile, several clinical studies have suggested that men subjects are more likely to have detectable coronary calcification than women [52], and coronary atherosclerosis in men tends to be more associated with calcification than similarly affected arteries in women [52]. Since the effect of vitamin D on subclinical atherosclerosis may be different according to gender, optimal range of serum vitamin D to prevent subclinical atherosclerosis should be decided by considering gender differences. These results warrant further studies on the specified population groups according to menopause or andropause to ascertain the gender-related difference in effect of vitamin D on CAC in more detail.

Several limitations of our study should be mentioned. First, since this study was cross-sectionally designed, we couldn’t propose cause-and-effect relationships. In the future, a prospective study will be needed to validate the findings in our study. Second, our results were obtained in a relatively healthy occupational cohort, so the application of our results cannot be extended to other populations. Third, an experimental study should be conducted to support the mechanism for the potentially harmful effect of vitamin D on CAC. Fourth, we were unable to adjust for sunlight exposure, dietary factor and vitamin D supplementation which are important influencing factors on vitamin D status. Instead of sunlight exposure, we used data for season as a adjustment factor to compliment this limitation. Fifth, the data might be underpowered due to small sample size of women with CAC > 0. Finally, we couldn’t analyze subgroups according to menopause or andropause due to lack of data, although these may influence the status of vitamin D and metabolic diseases. Despite these limitations, our study has important strengths that differentiate it from previous studies. We used data from a large sample size with homogenous characteristics like relatively healthy young Korean adults and considered various potential confounding factors such as age, smoking, alcohol, education, physical activity, season of year, and so on. Also, we analyzed data that allowed analysis according to gender. To our knowledge, this is the first study that reports a positive association between vitamin D and CAC in Koreans men.

## Conclusion

In summary, the present study suggests that high levels of serum vitamin D were associated with a lower risk of MS, IR and FL in both Korean men and women, but were associated with a higher risk of CAC only in men, and not in women.

## Additional file

10.1186/s12933-016-0432-3 General characteristics by quartiles of serum vitamin D levels in men with CAC data (n = 19,999). **Table S2.** General characteristics by quartiles of serum vitamin D levels in women with CAC data (n = 5510).

## Abbreviations

CAC: coronary artery calcification
CVD: cardiovascular disease
DBP: diastolic blood pressure
DM: diabetes mellitus
FL: fatty liver
HDL-C: HDL cholesterol
HTN: hypertension
IR: insulin resistance
hs-CRP: high sensitive C-reactive protein
MS: metabolic syndrome
SBP: systolic blood pressure
TC: total cholesterol
TG: triglyceride
