# Supplementary material for: High levels of serum vitamin D are associated with a decreased risk of metabolic diseases in both men and women, but an increased risk for coronary artery calcification in Korean men
Source: Cardiovasc Diabetol. 2016 Aug 12;15:112. doi: 10.1186/s12933-016-0432-3 (PMC4983097; doi:10.1186/s12933-016-0432-3)
Supplement: Supplementary file 1 — 10.1186/s12933-016-0432-3 General characteristics by quartiles of serum vitamin D levels in men with CAC data (n = 19,999). Table S2. General characteristics by quartiles of serum vitamin D levels in women with CAC data (n = 5510). [file 12933_2016_432_MOESM1_ESM.docx]

Table S1: General characteristics by quartiles of serum vitamin D levels in men with CAC data (n=19,999)

| **Characteristics** | **Total subjects** | | **Quartiles of Serum Vitamin D levels** | | | | ***P* for trend** |
| --- | --- | --- | --- | --- | --- | --- | --- |
|  |  |  | **Q1**  **(<13.0mg/dl)** | **Q2**  **(13-16.8mg/dl)** | **Q3**  **(16.8-21.5mg/dl)** | **Q4**  **(≥21.5mg/dl)** |  |
|  |  | | **n=5,006** | **n=4,994** | **n=5,000** | **n=4,999** |  |
| Age (yrs) | | 40.1 (7.4) | 38.4 (6.8) | 39.4 (7.0) | 40.4 (7.1) | 42.2 (8.0) | <0.001 |
| BMI (kg/m^2^) | | 24.8 (3.0) | 24.7 (3.2) | 24.8 (3.0) | 24.9 (3.0) | 24.8 (2.8) | 0.080 |
| Current smoker (%) | | 33.8 | 29.3 | 31.6 | 35.9 | 38.5 | <0.001 |
| Alcohol intake (%)^1)^ | | 33.1 | 25.2 | 31.5 | 35.3 | 40.5 | <0.001 |
| Regular exercise (%)^2)^ | | 13.9 | 10.7 | 12.6 | 14.8 | 17.5 | <0.001 |
| Higher education (%)^3)^ | | 87.7 | 89.2 | 89.0 | 87.6 | 84.9 | <0.001 |
| SBP (mmHg) | | 113.4 (11.5) | 113.2 (11.4) | 113.4 (11.6) | 113.4 (11.3) | 113.4 (11.6) | 0.330 |
| DBP (mmHg) | | 74.4 (9.6) | 74.1 (9.6) | 74.4 (9.7) | 74.5 (9.5) | 74.7 (9.5) | 0.001 |
| Glucose (mg/dl) | | 98.2 (16.2) | 97.5 (15.7) | 97.6 (15.5) | 98.6 (17.0) | 99.1 (16.6) | <0.001 |
| Insulin (μU/mL)^4)^ | | 6.13 (4.16-8.85) | 6.4 (4.4-9.31) | 6.21 (4.17-8.96) | 6.17 (4.13-8.85) | 5.77 (3.94-8.35) | <0.001 |
| TC (mg/dl) | | 202.5 (35.1) | 199.7 (34.7) | 202.9 (36.0) | 204.2 (35.2) | 203.1 (34.5) | <0.001 |
| LDL-C (mg/dl) | | 129.8 (31.8) | 127.4 (31.2) | 130.1 (32.2) | 131.5 (32.0) | 130.2 (31.4) | <0.001 |
| HDL-C (mg/dl) | | 51.4 (12.2) | 50.5 (12.0) | 51.2 (12.0) | 51.5 (12.2) | 52.5 (12.5) | <0.001 |
| TG (mg/dl)^4)^ | | 121 (86-173) | 121 (85-175) | 121 (86-172) | 122 (87-174) | 121 (86-172) | 0.223 |
| hs-CRP (mg/L)^4)^ | | 0.6 (0.3-1.1) | 0.6 (0.3-1.1) | 0.6 (0.3-1.1) | 0.6 (0.3-1.1) | 0.6 (0.3-1.1) | 0.744 |
| Obesity (%) | | 43.5 | 41.3 | 44.3 | 44.8 | 43.5 | 0.025 |
| Abdominal obesity (%) | | 33.6 | 33.4 | 34.8 | 34.0 | 32.2 | 0.119 |
| DM (%) | | 5.23 | 4.10 | 4.79 | 5.56 | 6.48 | <0.001 |
| HTN (%) | | 17.1 | 15.6 | 16.4 | 17.4 | 19.0 | <0.001 |
| FL (%) | | 46.8 | 47.9 | 47.9 | 47.5 | 44.1 | <0.001 |
| MS (%) | | 17.2 | 17.3 | 17.3 | 17.4 | 17.0 | 0.743 |
| HOMA-IR^4)^ | | 1.46 (0.97-2.18) | 1.53 (1.02-2.27) | 1.46 (0.97-2.20) | 1.48 (0.97-2.18) | 1.39 (0.93-2.09) | <0.001 |

Data are expressed as mean (SD), 1): ≥ 20g/day, 2): ≥ 3 times/week, 3): ≥ college graduate, 4) Data are expressed as median (IQR), BMI: body mass index, SBP: systolic blood pressure, DBP: diastolic blood pressure, TC: total cholesterol, HDL-C: HDL cholesterol, TG: triglyceride, hs-CRP: high sensitive C-reactive protein, DM: diabetes mellitus, HTN: hypertension, FL: Fatty liver MS: metabolic syndrome, CVD: cardiovascular disease, HOMA-IR: Homeostasis Model Assessment of Insulin Resistance

Table S2: General characteristics by quartiles of serum vitamin D levels in women with CAC data (n=5,510)

| **Characteristics** | **Total subjects** | | **Quartiles of Serum Vitamin D levels** | | | | ***P* for trend** |
| --- | --- | --- | --- | --- | --- | --- | --- |
|  |  |  | **Q1**  **(<9.8mg/dl)** | **Q2**  **(9.8-13.0mg/dl)** | **Q3**  **(13.0-17.6mg/dl)** | **Q4**  **(≥17.6mg/dl)** |  |
|  |  | | **n=1,380** | **n=1,378** | **n=1,378** | **n=1,374** |  |
| Age (yrs) | | 41.8 (9.6) | 40.9 (9.2) | 40.9 (9.3) | 41.6 (9.3) | 43.7 (10.3) | <0.001 |
| BMI (kg/m^2^) | | 22.4 (3.3) | 22.4 (3.5) | 22.5 (3.4) | 22.4 (3.2) | 22.2 (3.0) | 0.055 |
| Current smoker (%) | | 1.79 | 2.57 | 1.42 | 1.61 | 1.56 | 0.108 |
| Alcohol intake (%)^1)^ | | 5.70 | 4.41 | 6.04 | 6.63 | 5.71 | 0.137 |
| Regular exercise (%)^2)^ | | 14.6 | 11.4 | 13.2 | 14.9 | 18.7 | <0.001 |
| Higher education (%)^3)^ | | 67.2 | 65.1 | 64.5 | 70.8 | 68.6 | 0.004 |
| SBP (mmHg) | | 102.4 (12.2) | 102.5 (12.1) | 102.4 (12.3) | 102.2 (12.3) | 102.3 (12.0) | 0.527 |
| DBP (mmHg) | | 66.0 (9.2) | 66.2 (9.1) | 65.9 (9.2) | 66.0 (9.3) | 66.0 (9.1) | 0.646 |
| Glucose (mg/dl) | | 93.5 (14.1) | 93.7 (13.6) | 93.7 (14.8) | 93.5 (15.0) | 93.0 (12.7) | 0.205 |
| Insulin (μU/mL)^4)^ | | 4.97 (3.41-7.21) | 5.11 (3.52-7.62) | 5.11 (3.52-7.51) | 4.98 (3.33-7.01) | 4.69 (3.22-6.67) | <0.001 |
| TC (mg/dl) | | 193.8 (34.8) | 190.6 (33.4) | 194.5 (36.2) | 194.1 (35.0) | 195.8 (34.5) | <0.001 |
| LDL-C (mg/dl) | | 116.7 (32.0) | 114.5 (31.0) | 117.3 (33.3) | 116.9 (31.9) | 118.0 (31.6) | 0.009 |
| HDL-C (mg/dl) | | 62.6 (14.9) | 61.1 (14.6) | 62.5 (14.7) | 63.0 (14.7) | 63.8 (15.5) | <0.001 |
| TG (mg/dl)^4)^ | | 76 (57-108) | 78 (58-110) | 75(56-106) | 76.5 (57-109) | 75 (57-105) | 0.338 |
| hs-CRP (mg/L)^4)^ | | 0.4 (0.2-0.8) | 0.3 (0.2-0.7) | 0.3 (0.2-0.8) | 0.4 (0.2-0.8) | 0.4 (0.2-0.8) | 0.094 |
| Obesity (%) | | 18.4 | 19.2 | 19.9 | 18.3 | 16.0 | 0.014 |
| Abdominal obesity (%) | | 35.0 | 34.3 | 35.5 | 34.9 | 35.1 | 0.745 |
| DM (%) | | 3.68 | 3.12 | 3.48 | 3.77 | 3.93 | 0.222 |
| HTN (%) | | 10.5 | 10.3 | 10.1 | 10.7 | 11.0 | 0.491 |
| FL (%) | | 18.0 | 18.0 | 18.5 | 19.0 | 16.5 | 0.383 |
| MS (%) | | 9.8 | 10.5 | 10.4 | 9.2 | 9.2 | 0.174 |
| HOMA-IR^4)^ | | 1.13 (0.75-1.69) | 1.16 (0.78-1.77) | 1.17 (0.78-1.77) | 1.12 (0.74-1.66) | 1.06 (0.71-1.58) | <0.001 |

Data are expressed as mean (SD), 1): ≥ 20g/day, 2): ≥ 3 times/week, 3): ≥ college graduate, 4) Data are expressed as median (IQR), BMI: body mass index, SBP: systolic blood pressure, DBP: diastolic blood pressure, TC: total cholesterol, HDL-C: HDL cholesterol, TG: triglyceride, hs-CRP: high sensitive C-reactive protein, DM: diabetes mellitus, HTN: hypertension, FL: Fatty liver MS: metabolic syndrome, CVD: cardiovascular disease, HOMA-IR: Homeostasis Model Assessment of Insulin Resistance
